# Exposure to artificial light at night and risk of cancer: where do we go from here?

**DOI:** 10.1038/s41416-020-01231-7

**Published:** 2021-01-22

**Authors:** Rena R. Jones

**Affiliations:** Occupational and Environmental Epidemiology Branch, Division of Cancer Epidemiology and Genetics, National Cancer Institute, National Institutes of Health, 9609 Medical Center Drive, Rockville, MD 20895 USA

**Keywords:** Risk factors, Oncology

## Abstract

Despite experimental and mechanistic data suggesting circadian disruption’s role in carcinogenesis, mixed findings from epidemiological investigations of artificial light at night and cancer risk in the general population are difficult to interpret due to exposure assessment limitations. It will be important for future studies to assess and validate individual-level exposures, ideally over the lifetime.

## Main

Exposure to artificial light at night (LAN) is commonplace in the developed world. LAN suppresses the production of melatonin, a critical regulator of circadian rhythms.^1^ Circadian disruption has been suggested as a potential mechanism for the development of several cancers, especially those for which hormone dysregulation is important. It may affect biological pathways involved in cancer carcinogenesis, such as hormone signalling, cell proliferation, DNA repair, and inflammation pathways.^1,2^ Night shift work has been classified by the International Agency for Research on Cancer as a probable human carcinogen based on sufficient evidence of cancer and strong mechanistic evidence in experimental animals, and limited epidemiological evidence, including for breast, prostate, and colorectal cancers.^3^ Animal data support the carcinogenicity of alterations in the light–dark schedule, which commonly occurs in shift workers. However, it is unknown whether general population exposure to LAN leads to perturbations in circadian function comparable with those demonstrated from night shift work, and how that may relate to cancer risk.

Multiple epidemiological studies have evaluated levels of both outdoor and indoor LAN and cancer, most consistently observing increased risk for breast cancer associated with high exposure;^4–6^ however, some found no overall association.^7,8^ In our study among postmenopausal women in a large U.S. cohort, we observed a 10% increased risk of breast cancer for women in the highest versus the lowest quintile of outdoor LAN exposure,^9^ an effect similar in magnitude to those reported by other cohorts. Our results suggested a stronger effect among individuals with oestrogen-receptor-positive tumours; this finding is consistent with several other studies, but most subtype evaluations have been limited in power.^5–7^ Associations in our study also appeared to vary by other individual characteristics such as smoking, alcohol consumption, sleep duration, body mass index, and neighbourhood environment; however, there is little consistency in these subgroup effects across studies.

The greatest weakness of observational studies of LAN and cancer is the exposure assessment, which often lacks the temporal and spatial resolution in LAN measurements necessary for adequate exposure specificity. These studies consequently suffer from exposure misclassification that is expected to be non-differential with respect to outcome, thus attenuating observed risk estimates. Studies investigating LAN associations in the general population typically have relied on exposure proxies, including external measurements assessed at large geographic scales (e.g., satellite imagery) and to a lesser extent, self-reported surveys. Few studies have characterised the frequency and duration of these exposures over a long period of time including periods relevant for cancer development, nor have they characterised the physiologically relevant components of light exposure.

The most common method to assess LAN relies on measurements of ground-level outdoor illumination from satellite images taken at night. These measures are typically at a large spatial scale (~1 km) and previous studies have demonstrated their low correlation with indoor LAN measurements,^10^ indicating they may poorly reflect personal exposure. Another limitation is that radiance measures from satellites capture light intensity but do not provide information about the full spectrum of night-time light emissions. The biological responses to light exposure are dependent on the light intensity, duration and wavelength,^1^ but few cancer studies have evaluated these components of LAN. Two recent evaluations of outdoor LAN in a multi-site case–control study in Spain, the first cancer study to use spectral information from satellite images to differentiate light wavelengths, suggested that light in the blue spectrum, rather than total visible light, is associated with increased risks of breast, prostate^11^ and colorectal^12^ cancers. These findings have important implications because of the increasing blue light exposure in the general population concomitant with the use of electronic devices with screens. Blue light exposure has been shown to more strongly suppress circadian rhythms and melatonin levels compared with the light of other wavelengths.^13^ Indoor LAN is arguably a better indicator of individual-level exposure than outdoor measures. However, indoor light exposures captured through self-reported data have often led only to semi-quantitative assessments, such as the amount of time spent in an illuminated room. Few validations of these exposures have been conducted, especially over a substantial portion of an individual’s lifetime. Increasingly, devices such as photometers or data loggers that track indoor light usage have been applied in observational studies of non-cancer endpoints, including melatonin levels, insomnia and sleep latency, obesity and depression.^13^ However, no published studies of cancer have incorporated these devices. Measuring light exposure on every individual in a study population may not be feasible, and is not possible retrospectively, therefore exposure studies are needed to better characterise personal LAN exposures and determinants of exposure over various time periods for use in historical exposure assessments.

The majority of LAN studies have lacked information on important contextual factors that may influence total artificial light exposure, including night-time activities both indoors and outdoors, window treatments, and illumination from specific lights and devices in the home. Moreover, given the potential correlation between LAN and characteristics of the urban environment that may also relate to cancer risk, inadequate control for confounding is a concern in these studies. Most have attempted to account for these factors (e.g., outdoor air pollution, socioeconomic status) through covariate adjustment in risk models, but it remains unclear whether observed relationships with LAN are due to residual confounding.

Most evaluations of the relationship between LAN exposure and cancer among the general population are subject to misclassification of exposure, which may explain the mixed associations observed across studies with varying quality of exposure assessment. Artificial light exposure is a modifiable risk factor, and thus better understanding of the association with cancer incidence has important implications for public health prevention efforts. Improved exposure characterisation over aetiologically relevant time periods is needed to clarify these relationships.

## Data Availability

Not applicable.
